# Aqueous humor levels of TGFβ2 and SFRP1 in different types of glaucoma

**DOI:** 10.1186/s12886-019-1183-1

**Published:** 2019-08-05

**Authors:** Tao Guo, Li Guo, Yuchen Fan, Li Fang, Jiahong Wei, Ye Tan, Yuhong Chen, Xianqun Fan

**Affiliations:** 1 Department of Ophthalmology Shanghai Ninth People’s Hospital, Shanghai JiaoTong University School of Medicine, Shanghai, 200011 China; 2 Shanghai Key Laboratory of Orbital Diseases and Ocular Oncology, Shanghai, 200011 China; 3 Bengbu Medical College, Bengbu, 233030 Anhui province China; 4 Department of Ophthalmology, Luan Affiliated Hospital of Anhui Medicine University, Luan, 237000 Anhui province China; 5 Department of Ophthalmology, Shanghai Pudong District Gongli Hospital, Shanghai, 201201 China; 6 Department of Ophthalmology Eye & ENT Hospital Shanghai Medical College, Fudan University, Shanghai, 200031 China

**Keywords:** Transforming growth factor-β2, Secreted frizzled-related protein-1, Aqueous humor, Glaucoma, Intraocular pressure

## Abstract

**Background:**

To assess bioactive transforming growth factor-β2 (TGFβ2) and secreted frizzled-related protein-1 (SFRP1) levels in aqueous humor (AH) of different types of glaucoma.

**Methods:**

AH samples were obtained immediately before ophthalmic surgery with a 27-gauge needle attached to a microsyringe from 126 eyes (105 patients) divided into five groups: cataract (control), primary open-angle glaucoma (POAG), chronic angle-closure glaucoma (CACG), primary angle-closure suspects (PACS), and acute angle-closure glaucoma (AACG). Bioactive TGFβ2 and SFRP1 levels were assayed by ELISA.

**Results:**

The concentration of TGFβ2 in AH of POAG patients, but not CACG, PACS, or AACG patients, was significantly higher than control eyes. However, within the AACG group, although the TGFβ2 levels in AH did not differ significantly from the control level when all AACG patients were grouped together, there were differences when the AACG patients were divided into high and normal intraocular pressure (IOP); TGFβ2 of AACG patients with high IOP (> 21 mmHg) was significantly higher than those with normal IOP. AH levels of SFRP1 were not significantly different among the groups. However, a statistical significant, negative correlation between SFRP1 and IOP existed in the POAG group. POAG patients with high IOP had lower levels of SFRP1 than those with normal IOP. In contrast, a significant, positive correlation between SFRP1 level and IOP was detected in the AACG group. AACG patients with high IOP had a higher level of SFRP1 than those with normal IOP. Concentrations of TGFβ2 and SFRP1 did not correlate significantly with each other, or with age.

**Conclusions:**

These results indicate that AH levels of TGFβ2 and SFRP1 showed different profiles in different types of glaucomas.

**Electronic supplementary material:**

The online version of this article (10.1186/s12886-019-1183-1) contains supplementary material, which is available to authorized users.

## Background

Glaucoma is a leading cause of irreversible blindness in the world. According to the anatomical shape of the anterior chamber angle, glaucoma can be divided into open-angle glaucoma (OAG) and angle-closure glaucoma (ACG). It is projected that primary open angle glaucoma (POAG) will affect 53 million people while primary angle-closure glaucoma (PACG) will affects 23 million worldwide in 2020 [1]. In PACG patients, the outflow pathway of aqueous humor (AH) is obstructed anatomically by apposition of the iris [2], whereas in POAG, ocular hypertension is a result of increased AH outflow resistance in the trabecular meshwork (TM), which is likely associated with increased local deposition of extracellular matrix and stiffening of the tissue [3–5].

The exact mechanism of pathogenesis in glaucoma is not known. However, elevated levels of several cytokines, such as transforming growth factor β2 (TGFβ2) and secreted frizzled-related protein 1 (SFRP1), have been detected in the AH and/or TM of POAG patients. For example, TGFβ2 levels are higher in AH samples of POAG patients [6–15]. TGFβ2 is produced by tissues in the anterior segment of eye and affects TM cell functions, such as extracellular matrix metabolism, contractility, phagocytosis and proliferation [16–22]. The cellular functions of TGFβ2 likely translate to elevation of intraocular pressure (IOP). Perfusion of ex vivo human anterior segments with TGFβ2 increases IOP, corresponding to an increased accumulation of extracellular matrix in the TM region [17, 23]. Most importantly, intraocular injection of an adenoviral vector encoding a biologically active form of TGFβ2 increases IOP in both the rat and mouse [24–26].

SFRP1 is an antagonist of the Wnt signaling pathway, which has been localized in many cells and tissues [27], including the human TM [28]. By preventing Wnt from activating its receptor, SFRP1 blocks the Wnt effects on normal cellular functions and decreases the intracellular level of catenin. In cultured TM cells derived from POAG patients, SFRP1 expression was significantly higher than non-glaucoma controls, concomitant with a decrease in catenin level [29]. Treatment of ex vivo perfused human anterior segments with recombinant SFRP1 reduced AH outflow facility [29]. Intravitreal injection of adenoviral vector encoding SFRP1 increases ocular expression of the protein and raises IOP in the mouse [28, 29], clearly indicating a potential contributory role in glaucoma. However, SFRP1 levels in AH of glaucoma patients have not been reported.

Although these two cytokines are elevated in TM tissues of POAG patients, it is not clear whether the two proteins are simultaneously increased in the same patients and if their levels in AH samples of ACG patients are changed. It is also not known if the concentrations of these two cytokines correlate with IOP. To attempt to address these questions, we evaluated the two cytokines levels in AH of POAG and ACG patients to investigate the involvement of these cytokines in glaucomas.

## Materials and methods

### Patient and sample collection

This study protocol was approved by the Research Ethics Committee of the Shanghai Ninth People’s Hospital, Shanghai JiaoTong University School of Medicine, and adhered to the tenets of the Helsinki Declaration. All patients provided written informed consent.

The AH samples were collected from 126 eyes of 105 patients, who were divided into the following groups: cataract patients who did not have any other known ocular disorders (*n* = 26 eyes of 24 patients; serving as controls), POAG (*n* = 25 eyes of 21 patients), chronic ACG (CACG) (*n* = 21 eyes of 19 patients), primary angle-closure suspects (PACS) (*n* = 9 eyes of 9 patients), and acute ACG (AACG) (*n* = 45 eyes of 32 patients).

All patients underwent careful and detailed ophthalmologic evaluations, including anterior chamber angle, visual field, optic disc morphology and IOP. All patients had no history of trabeculectomy or laser iridotomy surgeries. POAG was defined as the presence of glaucomatous optic nerve damage, visual field loss and normal angle on gonioscopy. CACG was defined as the existence of a gonioscopically occludable angle accompanied by glaucomatous optic neuropathy and compatible visual field defects. Eyes with an occludable drainage angle, but normal optic disc, visual field, IOP, and no peripheral anterior synechiae were diagnosed as PACS. The following criteria were used to define cases of AACG: (1) presence of at least two of the following symptoms: ocular or periocular pain, nausea or vomiting, headache, an antecedent history of intermittent blurring of vision with haloes; (2) presenting of an occluded angle on gonioscopy and IOP > 21 mmHg; (3) the presence of at least three of the following signs: conjunctival injection, corneal epithelial edema, mid-dilated unreactive pupil, and shallow anterior chamber.

Cataract and PACS patients did not receive any ophthalmic medication within one month prior to AH collection. All patients of the POAG and CACG groups were on anti-glaucoma eye drops that included beta-blockers, carbonic anhydrase inhibitors, and alpha-adrenergic agonists. In the AACG group, most patients received the same medications, except for eight patients, who experienced their first AACG attack with high IOP (> 40 mmHg). AH samples of these eight patients were collected while they were treated by anterior chamber puncture. AH samples of other patients in our study were collected by at the time of cataract or glaucoma surgery after medical treatment. The patients underwent IOP measurement by a Goldmann applanation tonometer within half an hour before AH sample collection. Within each of the POAG, CACG, and AACG groups, for additional analysis, we further divided the patients into two sub-groups, those whose IOP values were managed by therapeutic means to ≤21 mmHg, and those whose IOP were > 21 mmHg. Characterizations of the patients in each group are listed in Table 1.

**Table 1:** Clinical characteristics of patients

Diagnosis	Sample size	Male	Female	Age (y,mean ± SEM)	IOP (mmHg, mean ± SEM)
Cataract	26	8	18	67.5 ± 1.7	12.6 ± 0.6
POAG	25	18	7	66.2 ± 2.0	22.1 ± 2.2
CACG	21	10	11	71.2 ± 1.9	25.3 ± 1.8
PACS	9	1	8	73.0 ± 2.4	12.4 ± 1.3
AACG	45	11	34	62.7 ± 2.1	31.1 ± 2.4

Abbreviations: *IOP* intraocular pressure at the time of aqueous humor collection, *POAG* primary open-angle glaucoma, *CACG* chronic angle-closure glaucoma, *PACS* primary angle-closure suspects, *AACG* acute angle-closure glaucoma

Approximately 150 μL of AH was obtained at the time of anterior chamber puncture treatment and cataract or glaucoma surgery with a 27-gauge needle attached to a microsyringe. During AH collection, the needle was carefully positioned to avoid touching the corneal endothelium, iris, or lens. Free of blood contamination was visually confirmed. The samples were placed into Eppendorf tubes and stored at − 80 °C until analyzed.

### ELISA assays for TGFβ2 and SFRP1

Prior to assay, the thawed AH samples were centrifuged at 4 °C to remove potential debris. The concentration of the bioactive form of TGFβ2 in AH was measured by enzyme-linked immunosorbent assay (DB250, R&D Systems, Minneapolis, MN, USA). After adding 100 μL of ELISA assay diluent, 100 μL bioactive TGFβ2 standard or diluted AH samples were added to each well and incubated for 2 h at room temperature. Each well was washed 3 times with 400 μL wash buffer and 200 μL anti-TGFβ2 polyclonal antibody conjugated to horseradish peroxidase was added. The mixture was then incubated and washed. An aliquot of 200 μL substrate solution was added, and plates, protected from light, were incubated for 20 min at room temperature. Stop solution (50 μL) was then added to each well, and the absorbance at 450 nm was measured with a microplate reader (Pierce, Rockford, IL, USA).

The level of SFRP1 was also assayed by ELISA (ELH-SFRP1, RayBiotech, Norcross, GA, USA). Briefly, either 100 μL SFRP1 standard or diluted AH sample was added to each well. After 2.5 h incubation at room temperature with gentle shaking, the wells were washed four times with 300 μL buffer. Each well was coated with 100 μL of biotinylated anti-SFRP1 antibody and incubated for 1 h at room temperature. After being washed again, 100 μL stop solution was added to each well and the absorbance was measured similar to the TGFβ2 ELISA.

### Statistical analysis

Results are represented as mean ± SEM. Comparison among three or more groups was performed using one-way analysis of variance (ANOVA), followed by Dunnett’s test. Comparison between two groups was conducted by t-test. The correlation between two parameters was assessed by linear regression analysis. Statistical significance was set at *P* < 0.05 and all analyses were performed using SPSS 19.0.

## Results

### Levels of bioactive TGFβ2 and SFRP1 in AH

**Fig. 1:**
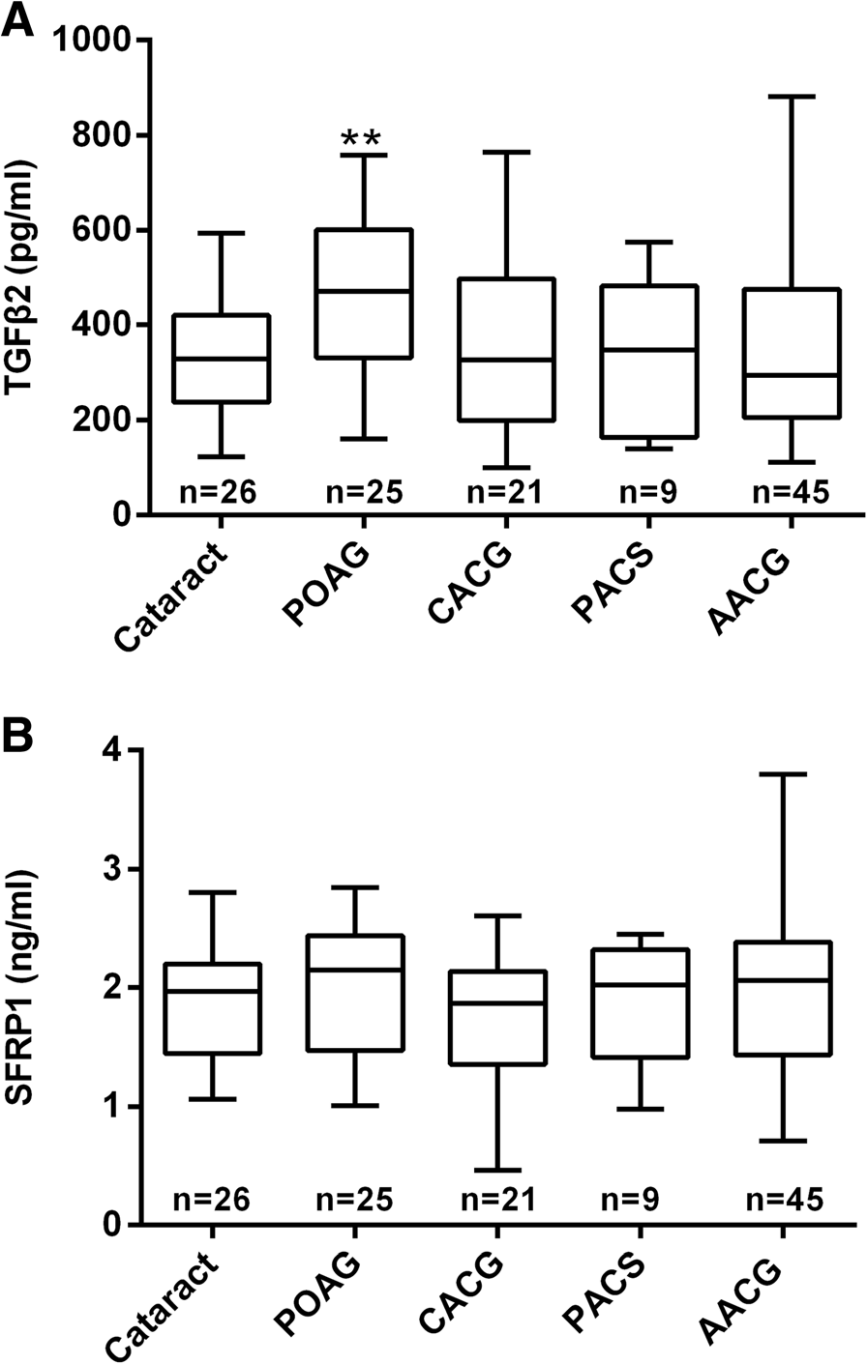
Aqueous humor concentrations of bioactive TGFβ2 (**a**) and SFRP1 (**b**) in different groups of patients. **: *p* < 0.01 vs. the Cataract (control) group by one-way ANOVA, followed by Dunnett’s test. Abbreviations: POAG = primary open-angle glaucoma, CACG = chronic angle-closure glaucoma, PACS = primary angle-closure suspects, AACG = acute angle-closure glaucoma

Our results show that the concentration of bioactive TGFβ2 in AH in control (cataract) eyes was 345.2 ± 24.8 pg/ml (mean ± SEM, *n* = 26) (Fig. 1a). This value corroborates very well with the majority of previously published reports (Additional file 1: Figure S1, [7–12, 14]. AH samples from POAG eyes had a bioactive TGFβ2 level of 465.9 ± 33.9 pg/ml (*n* = 25), which was significantly higher than that of control eyes (Fig. 1a) (*P* = 0.01). This finding again confirms prior observations that bioactive TGFβ2 level in AH samples of POAG patients are significantly higher than cataract controls [7–12, 14]. In comparison, there was no significant difference in concentrations of bioactive TGFβ2 between control eyes and CACG eyes (344.6 ± 37.4 pg/ml, *n* = 21) (*P* = 0.99), PACS eyes (336.0 ± 54.3 pg/ml, *n* = 9) (*P* = 0.89), or AACG (344.0 ± 26.5 pg/ml, *n* = 45) (*P* = 0.98) (Fig. 1a).

There were measureable levels of SFRP1 in AH samples. In cataract samples, a level of 1.88 ± 0.10 ng/ml was observed (Fig. 1b). Interestingly, SFRP1 levels in AH samples from patients with POAG (1.98 ± 0.12 ng/ml) (*P* = 0.54), CACG (1.71 ± 0.13 ng/ml) (*P* = 0.34), PACS (1.84 ± 0.18 ng/ml) (*P* = 0.85) and AACG (1.97 ± 0.10 ng/ml) (*P* = 0.53) were not significantly different from that of controls (Fig. 1b).

### Correlation between IOP and AH levels of TGFβ2 and SFRP1

**Fig. 2:**
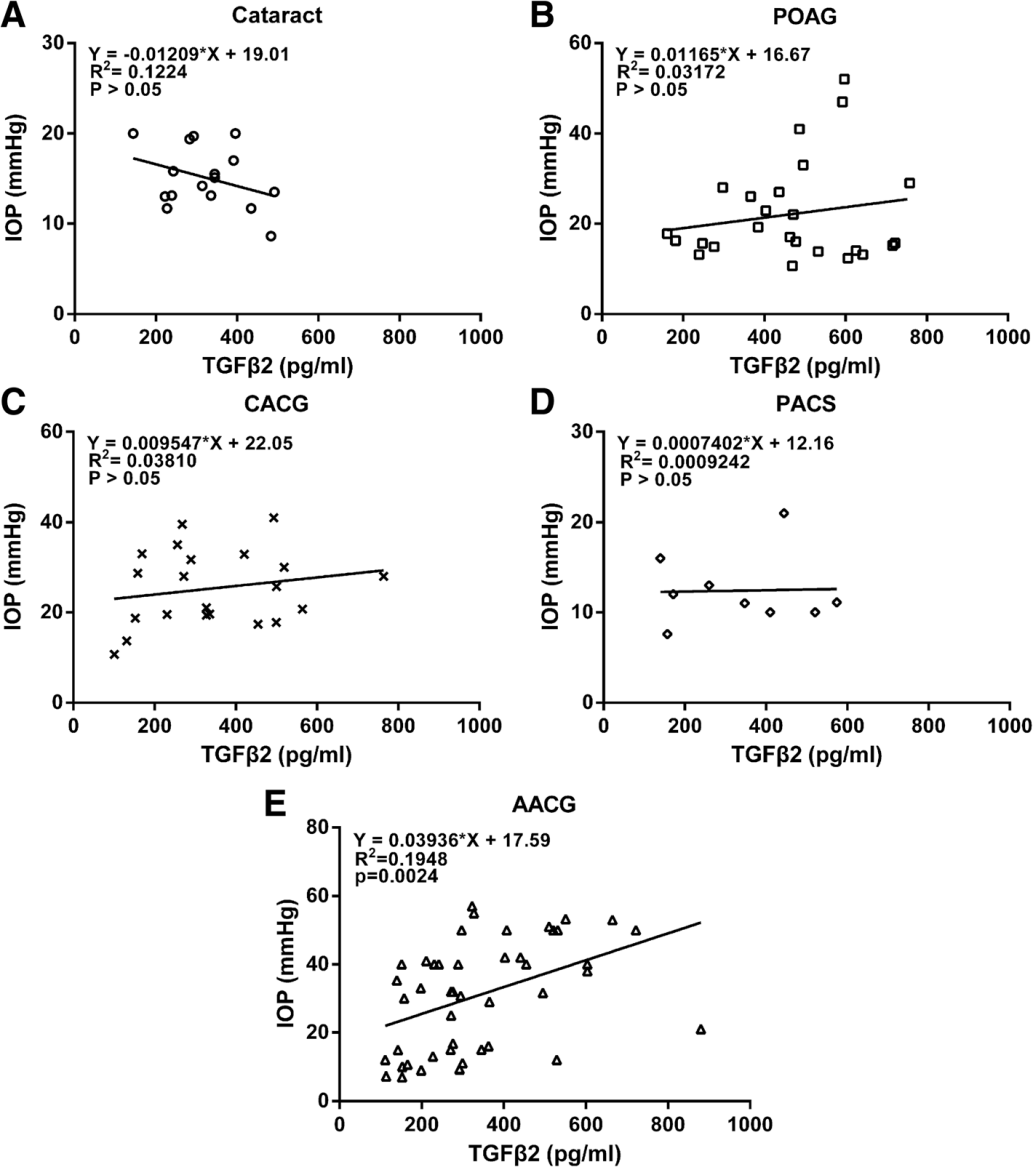
Correlation between IOP and the aqueous humor levels of TGFβ2 in Cataract (**a**), POAG (**b**), CACG (**c**), PACS (**d**) and AACG (**e**) groups of patients. Abbreviations: IOP = intraocular pressure, POAG = primary open-angle glaucoma, CACG = chronic angle-closure glaucoma, PACS = primary angle-closure suspects, AACG = acute angle-closure glaucoma

**Fig. 3:**
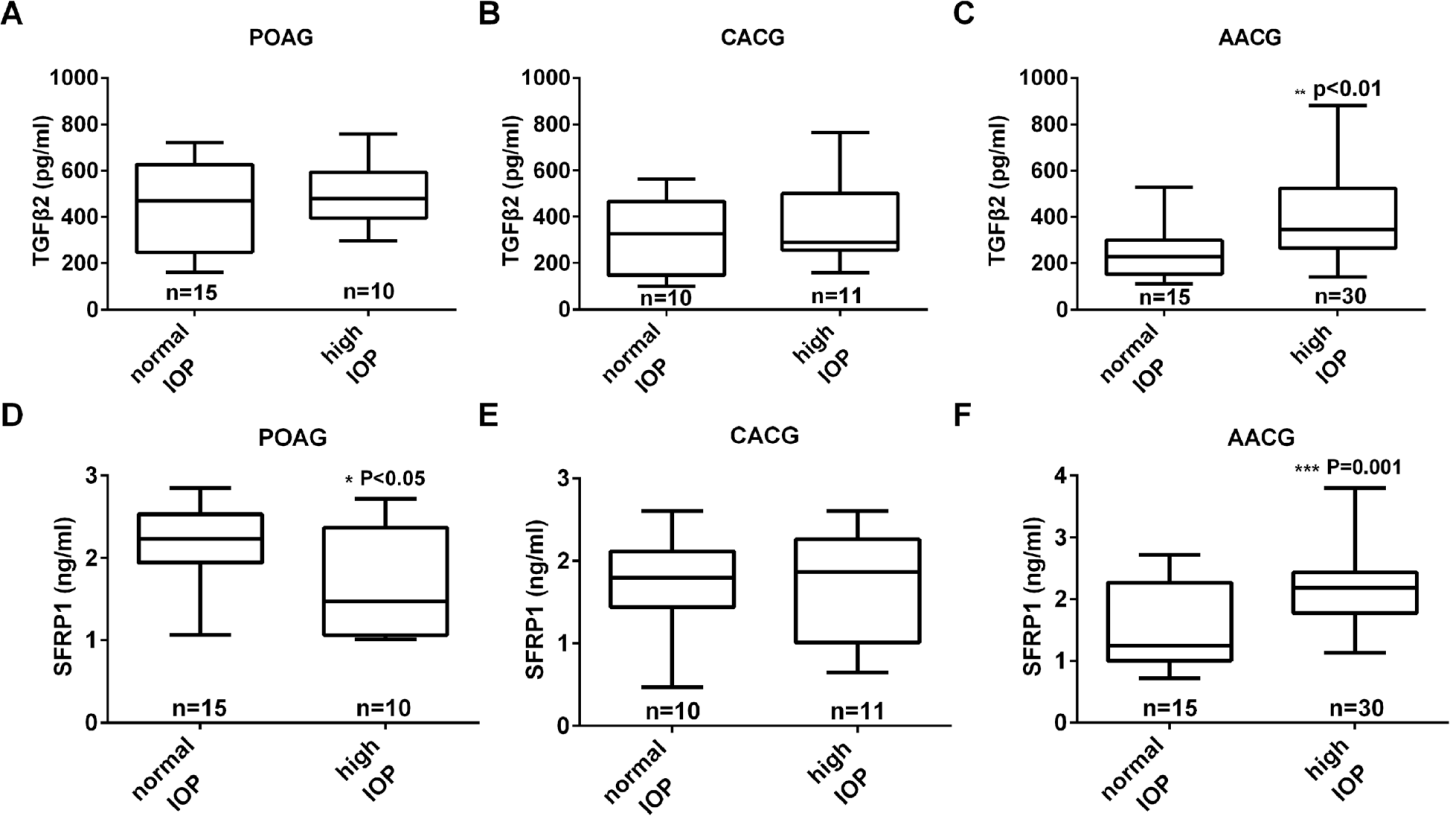
Comparisons of aqueous humor concentrations of bioactive TGFβ2 and SFRP1 in different sub-groups of patients. Aqueous humor concentrations of bioactive TGFβ2 in POAG (**a**), CACG (**b**) and AACG (**c**) groups of patients with high IOP (> 21 mmHg) and with normal IOP (≤ 21 mmHg). Aqueous humor concentrations of SFRP1 in POAG (**d**), CACG (**e**) and AACG(**f**) groups of patients with high IOP and with normal IOP. *: *p* < 0.05, **: *p* < 0.01, ***: *p* < 0.001 by t-test. Abbreviations: POAG = primary open-angle glaucoma, CACG = chronic angle-closure glaucoma, AACG = acute angle-closure glaucoma

To evaluate whether there was a correlation between IOP and the cytokines, we compared IOP within each disease group with the respective concentration of active TGFβ2 and SFRP1. The results indicate that within the control, POAG, PACS, and CACG groups, the IOP of the evaluated eyes at the time AH was collected did not correlate with TGFβ2 levels (*P* > 0.05), although a positive slope was observed in the POAG and CACG groups (Fig. 2a-d). Most importantly, a significant (*P* = 0.0024) correlation between the concentration of active TGFβ2 and IOP was detected in the AACG group (Fig. 2e). This finding led us to further divide the samples of this group into those whose IOP was successfully controlled to ≤21 mmHg (normal IOP), and those whose IOP was above 21 mmHg (high IOP). In doing so, the bioactive TGFβ2 levels were significantly (*P* = 0.006) different between these two groups: the level in “AACG with normal IOP” group was 242.8 ± 29.5 pg/ml (*n* = 15), and that in “AACG with high IOP” group was 394.6 ± 33.6 pg/ml (*n* = 30) (Fig. 3c). Using the same IOP criterion to divide samples in the POAG or CAGG groups into normal and high IOP sub-groups did not show significantly different TGFβ2 levels between the sub-groups (Fig. 3a, b).

**Fig. 4:**
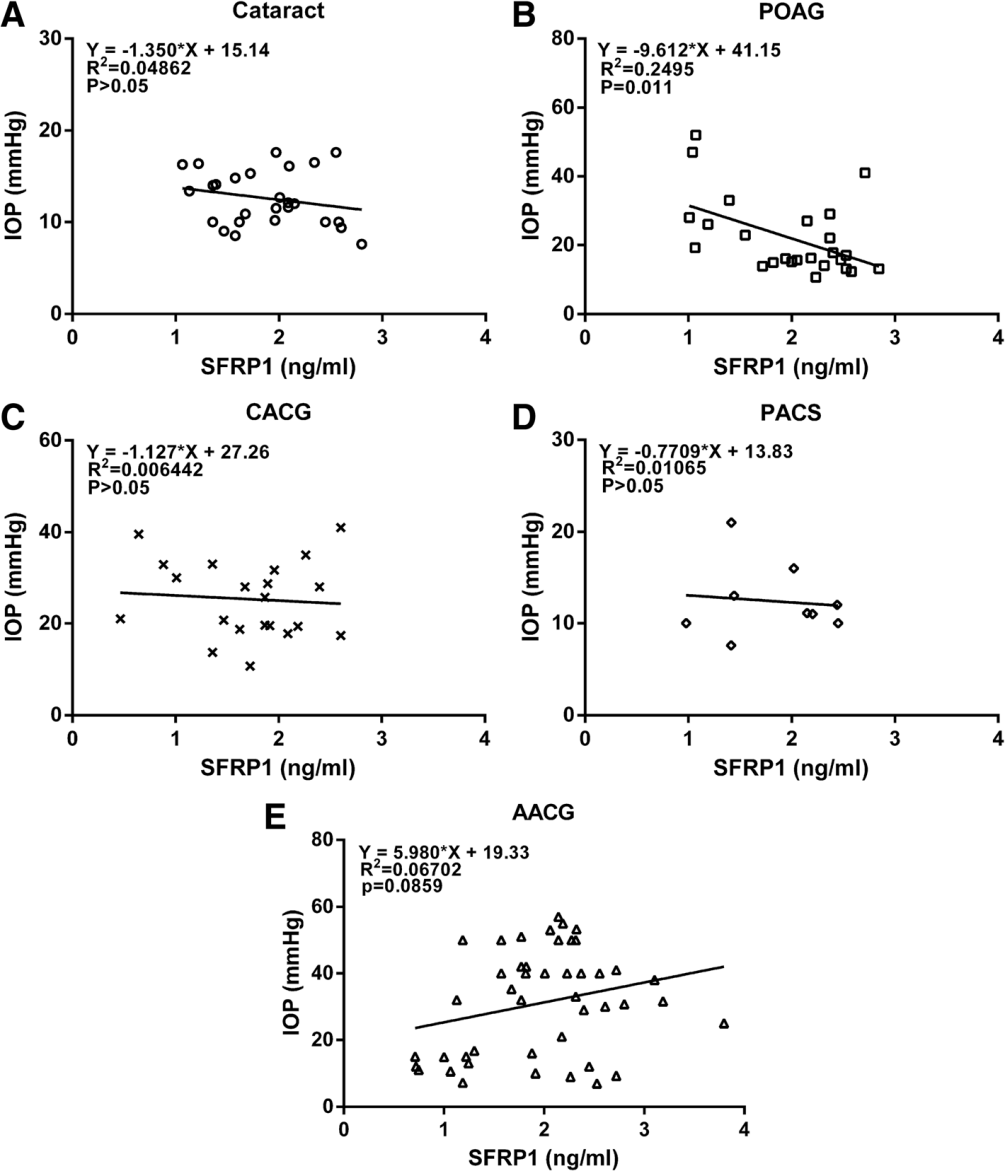
Correlation between IOP and the aqueous humor levels of SFRP1 in Cataract (**a**), POAG (**b**), CACG (**c**), PACS (**d**) and AACG (**e**) groups of patients. Abbreviations: IOP = intraocular pressure, POAG = primary open-angle glaucoma, CACG = chronic angle-closure glaucoma, PACS = primary angle-closure suspects, AACG = acute angle-closure glaucoma

When we evaluated the relationship between IOP and AH levels of SFRP1, we unexpectedly found a statistically significant, negative correlation between the SFRP1 level and IOP in the POAG group (*P* = 0.011) (Fig. 4b), while no significant (*P* > 0.05) correlation was observed between IOP and AH concentration of SFRP1 in cataract, PACS, AACG or CACG group (Fig. 4a, c, d, e). Interestingly, this correlation in the AACG group was positive and closed to being statistically significant (*P* = 0.0859) (Fig. 4e). Sub-dividing patients in POAG, AACG and CACG groups into those with normal IOP (≤ 21 mmHg) and those with high IOP (> 21 mmHg) confirmed these observations. The SFRP1 level in POAG with normal IOP (2.18 ± 0.11 ng/ml, *n* = 15) was significantly (*P* = 0.032) higher than that of the high IOP subgroup (1.69 ± 0.21 ng/ml, *n* = 10) (Fig. 3d). In the sub-groups of AACG patients, the relationship was reversed, such that the SFRP1 level in AACG with high IOP (2.19 ± 0.10 ng/ml, *n* = 30) was significantly (*P* = 0.001) lower than that of AACG with normal IOP (1.53 ± 0.18 ng/ml, n = 15) (Fig. 3f). In CACG patients, SFRP1 levels between normal and high IOP subgroups are not significantly different from each other (Fig. 3e).

### Correlation between medication, age and AH levels of TGFβ2 and SFRP1

**Fig. 5:**
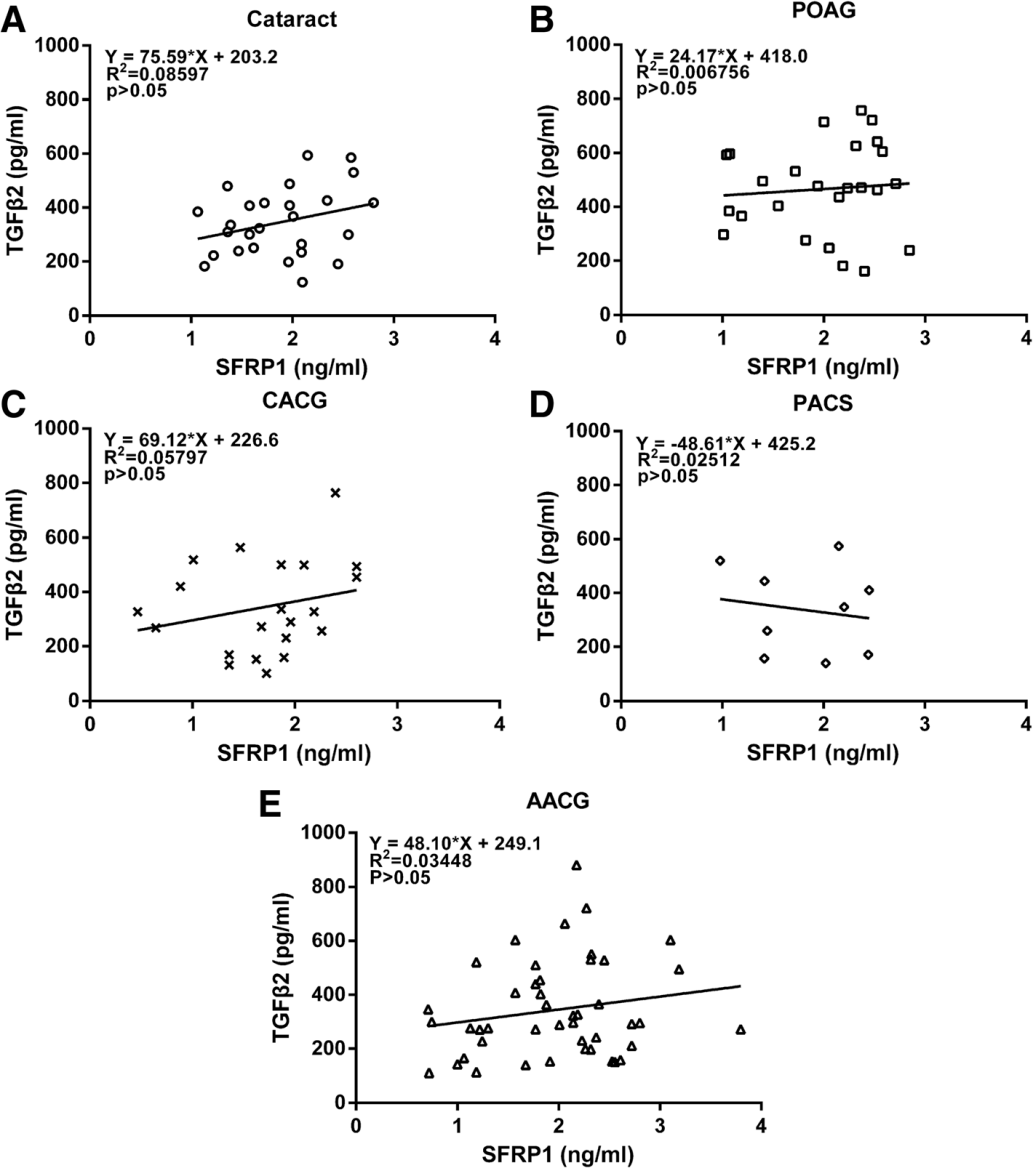
Lack of correlation between the AH levels of TGFβ2 and SFRP1 in Cataract (**a**), POAG (**b**), CACG (**c**), PACS (**d**) and AACG (**e**) groups of patients. POAG = primary open-angle glaucoma, CACG = chronic angle-closure glaucoma, PACS = primary angle-closure suspects, AACG = acute angle-closure glaucoma

To evaluate if changes of TGFβ2 and SFRP1 are associated with each other, we analyzed the correlation of levels between these two cytokines. There was no statistically significant correlation between the concentrations of active TGFβ2 and SFRP1 in the AH within each of the study groups (Fig. 5).

In the AACG group, 37 AH samples were obtained from patients treated with anti-glaucoma medication, and 8 samples were from patients who were not treated with medication. In order to analyze whether anti-glaucoma medications affected the experimental results, we divided AACG patients with high IOP into “drugs” group and “no drug” group, and found that there was not significantly difference in concentrations of bioactive TGFβ2 and SFRP1 between these two groups (Additional file 2: Figure S2). These findings suggest that anti-glaucoma medical treatment had no effect on the levels of TGFβ2 and SFRP1 within each group. The same analysis was not performed in the other groups, since all patients in the cataract and PACS groups did not receive any medication, and all patients in the POAG and CACG groups received anti-glaucoma eye drops.

We also analyzed the relationship between age and concentration of active TGFβ2 and SFRP1 levels in the AH. We did not detect any statistically significant correlation between age and the cytokine levels in any of the groups (data not shown).

## Discussion

Among other factors, TGFβ2 and SFRP1 have been implicated to be involved in glaucoma pathogenesis. In this study, we detected the presence of both TGFβ2 and SFRP1 in human AH samples. In cataract patients, the AH level of bioactive TGFβ2 was similar to many previously reported values [7–11, 14]. We also observed a significant elevation of active TGFβ2 in POAG patients, which is consistent with prior studies, although the magnitudes of changes were variable among the different studies [7–12, 14]. Previous studies reported no correlation between AH level of active TGFβ2 and IOP in POAG patients [8, 9], so did our findings.

In addition to POAG, our study evaluated TGFβ2 levels in PACG patients, divided into three groups: CACG, PACS and AACG. We found that the AH concentrations of active TGFβ2 in these groups were not significantly different from the cataract control group. However, when we assessed the correlation between IOP and TGFβ2 contents, we discovered a significant, positive correlation between these two parameters in the AACG group. This correlation was substantiated when the samples in the AACG group were sub-divided into patients whose IOP was controlled at ≤21 mmHg and those with high IOP. The TGFβ2 level in the high IOP sub-group was statistically significantly higher than that of the normal IOP sub-group. At this time, the biological and clinical significance of this finding is unclear. Nevertheless, we speculate that in the AACG patients with high IOP, there may have been inflammatory responses in the anterior segment of the eye, which may lead to an elevated level of TGFβ2 in the AH. Experiments are currently underway to test this hypothesis. Alternatively, a feed-forward relationship between ocular hypertension and TGFβ2 production may exist, as indicated by positive, though not significant, correlations in the POAG and CACG groups, such that a high IOP could augment AH level of TGFβ2. Since perfusion of ex vivo organ culture of human anterior segment with activated TGFβ2 increases IOP [17], and intraocular injection of adenoviral encoding the bioactive TGFβ2 causes ocular hypertension in both the rat and mouse [23], the increased TGFβ2 in AACG patients with high IOP may further exacerbate the glaucomatous conditions in the eye. Regardless, the contribution of TGFβ2 in ACG and clarification of these potential mechanisms deserve further careful evaluations.

We also measured the AH levels of SFRP1 in the same patients and found that they were not significantly different from that of the cataract control group. Wang and colleagues demonstrated that SFRP1 was overexpressed in TM tissues of POAG patients and in cultured TM cells derived from POAG donors [29]. They did not report the corresponding level in the AH. It is possible that because of the directional flow of AH, SFRP1 released in the TM quickly leaves the eye without adequate time for diffusion or equilibrium in the AH. Therefore, AH levels of SFRP1 do not reflect changes in the TM, and SFRP1 in AH likely comes from ocular tissues other than the TM.

Similar to TGFβ2, we found that SFRP1 concentration was elevated in AH samples in the AACG with high IOP sub-group. Since this cytokine is shown to be involved in inflammatory response, this further indicates that inflammation may play a role in AACG with high IOP. Wang and coworkers also showed that perfusion with recombinant SFRP1 decreases aqueous outflow in perfusion organ culture of human anterior segment [29]. And intravitreal injection of adenoviral vector encoding SFRP1 raises IOP in the mouse [29]. The source and contribution of elevated levels of SFRP1 in AACG with high IOP are currently unknown, the significance of these findings requires additional research.

Moreover, unexpectedly, the SFRP1 level in POAG with high IOP was lower than the normal IOP sub-groups. This regulation of SFRP1 expression is extremely intriguing. For the time being, the cause and the relationship to glaucoma pathogenesis of this finding are not apparent. It is possible that a feedback system exists, such that SFRP1 induces ocular hypertension, but high IOP reduces SFRP1 expression. This hypothesis will await future studies for clarification. We did not observe a difference between the high and normal IOP sub-groups in CACG patients.

In the AACG group, we did not observe significant differences in AH levels of TGFβ2 and SFRP1 between patients treated with anti-glaucoma medications and those who were not treated. This finding suggests that drug treatment did not affect the concentrations of the two cell factors in AH.

In addition, this study showed that there was no correlation between age and the two cytokines, suggesting that age is not a determining factor of expressions of these two factors. Furthermore, there was no correlation between TGFβ2 and SFRP1 in the same samples. It reveals that levels of the two cytokines in the different glaucoma groups are not synchronous, and each has its own expression profile. Clearly, they each is associated with their respective signaling pathway and exerts their specific effects on the regulation of IOP. It will be highly interesting to find out if their effects are additional, synergistic, or antagonistic.

There are several limitations in this study. Even though we detected statistical significance in certain comparisons, we feel that the sample sizes in these study groups were still relatively small. We did not further sub-divide the patient groups according to the glaucoma medications they received. In addition, because of limitation of AH sample amount, we were not able to evaluate other cytokines which may play a role in glaucoma.

## Conclusion

We detected TGFβ2 and SFRP1 in AH samples of the control and different glaucoma patients. We found that (1) POAG patients had a higher concentration of TGFβ2 in their AH, (2) AACG patients with high IOP had elevated levels of both cytokines compared to those with normal IOP, and (3) there was a difference in SFRP1 between normal and high IOP sub-groups in POAG patients. These results suggest that the relationships between glaucoma, IOP, and cytokine expression are very complicated. TGFβ2 and SFRP1 may play different roles in the pathogenesis and pathophysiology of different types of glaucoma. Glaucoma pathogenesis could be the result of many factors working together.

## Additional files

Additional file 1: **Figure S1.** Summary of previous published reports of bioactive TGFβ2 concentrations in aqueous humor of cataract and POAG patients. Data are shown as mean and SEM. POAG = primary open-angle glaucoma. (TIF 119 kb)

Additional file 2: **Figure S2.** In the AACG groups, 8 AH samples were collected from patients who did not received glaucoma medications (“no drug” group), while 37 AH samples were from patients who were treated with beta-blockers, carbonic anhydrase inhibitors, and alpha-agonists (labeled as “drugs”). No statistically significant difference (*p* > 0.05) was observed on levels of TGFβ2 and SFRP1 between these two groups. (TIF 43 kb)

## Data Availability

The datasets used and/or analysed during the current study available from the corresponding author on reasonable request.

## Abbreviations

AACG: Acute angle-closure glaucoma
ACG: Angle-closure glaucoma
AH: Aqueous humor
CACG: Chronic angle-closure glaucoma
IOP: Intraocular pressure
OAG: Open-angle glaucoma
PACS: primary angle-closure suspects
POAG: Primary open-angle glaucoma
SFRP1: Secreted frizzled-related protein-1
TGFβ2: Transforming growth factor-β2
TM: Trabecular meshwork

## References

[1] Tham YC, Li X, Wong TY, Quigley HA, Aung T, Cheng CY (2014). Global prevalence of glaucoma and projections of glaucoma burden through 2040: a systematic review and meta-analysis. Ophthalmology.

[2] Weinreb RN, Aung T, Medeiros FA (2014). The pathophysiology and treatment of glaucoma: a review. Jama.

[3] Tamm ER, Fuchshofer R (2007). What increases outflow resistance in primary open-angle glaucoma?. Surv Ophthalmol.

[4] Fuchshofer R, Tamm ER (2009). Modulation of extracellular matrix turnover in the trabecular meshwork. Exp Eye Res.

[5] Last JA, Pan T, Ding Y, Reilly CM, Keller K, Acott TS, Fautsch MP, Murphy CJ, Russell P (2011). Elastic Modulus determination of Normal and glaucomatous human trabecular meshwork. Invest Ophthalmol Vis Sci.

[6] Jampel HD, Roche N, Stark WJ, Roberts AB (1990). Transforming growth factor-beta in human aqueous humor. Curr Eye Res.

[7] Tripathi RC, Li J, Chan WF, Tripathi BJ (1994). Aqueous humor in glaucomatous eyes contains an increased level of tgf-beta 2. Exp Eye Res.

[8] Inatani M, Tanihara H, Katsuta H, Honjo M, Kido N, Honda Y (2001). Transforming growth factor-beta 2 levels in aqueous humor of glaucomatous eyes. Graefes Arch Clin Exp Ophthalmol.

[9] Picht G, Welge-Luessen U, Grehn F, Lutjen-Drecoll E (2001). Transforming growth factor beta 2 levels in the aqueous humor in different types of glaucoma and the relation to filtering bleb development. Graefes Arch Clin Exp Ophthalmol.

[10] Schlotzer-Schrehardt U, Zenkel M, Kuchle M, Sakai LY, Naumann GO (2001). Role of transforming growth factor-beta1 and its latent form binding protein in pseudoexfoliation syndrome. Exp Eye Res.

[11] Ochiai Y, Ochiai H (2002). Higher concentration of transforming growth factor-beta in aqueous humor of glaucomatous eyes and diabetic eyes. Jpn J Ophthalmol.

[12] Ozcan AA, Ozdemir N, Canataroglu A (2004). The aqueous levels of tgf-beta2 in patients with glaucoma. Int Ophthalmol.

[13] Yamamoto N, Itonaga K, Marunouchi T, Majima K (2005). Concentration of transforming growth factor beta2 in aqueous humor. Ophthalmic Res.

[14] Min SH, Lee TI, Chung YS, Kim HK (2006). Transforming growth factor-beta levels in human aqueous humor of glaucomatous, diabetic and uveitic eyes. Korean J Ophthalmol : KJO.

[15] Trivedi RH, Nutaitis M, Vroman D, Crosson CE (2011). Influence of race and age on aqueous humor levels of transforming growth factor-beta 2 in glaucomatous and nonglaucomatous eyes. J Ocul Pharmacol Ther.

[16] Fuchshofer R, Birke M, Welge-Lussen U, Kook D, Lutjen-Drecoll E (2005). Transforming growth factor-beta 2 modulated extracellular matrix component expression in cultured human optic nerve head astrocytes. Invest Ophthalmol Vis Sci.

[17] Fleenor DL, Shepard AR, Hellberg PE, Jacobson N, Pang IH, Clark AF (2006). Tgfbeta2-induced changes in human trabecular meshwork: implications for intraocular pressure. Invest Ophthalmol Vis Sci.

[18] Wordinger RJ, Clark AF (2007). Bone morphogenetic proteins and their receptors in the eye. Exp Biol Med (Maywood).

[19] Tovar-Vidales T, Clark AF, Wordinger RJ (2011). Transforming growth factor-beta2 utilizes the canonical smad-signaling pathway to regulate tissue transglutaminase expression in human trabecular meshwork cells. Exp Eye Res.

[20] Kang MH, Oh DJ, Kang JH, Rhee DJ (2013). Regulation of sparc by transforming growth factor beta2 in human trabecular meshwork. Invest Ophthalmol Vis Sci.

[21] Fuchshofer R, Tamm ER (2012). The role of tgf-beta in the pathogenesis of primary open-angle glaucoma. Cell Tissue Res.

[22] Wordinger RJ, Clark AF, Agarwal R, Lambert W, McNatt L, Wilson SE, Qu Z, Fung BK (1998). Cultured human trabecular meshwork cells express functional growth factor receptors. Invest Ophthalmol Vis Sci.

[23] Gottanka J, Chan D, Eichhorn M, Lutjen-Drecoll E, Ethier CR (2004). Effects of tgf-beta2 in perfused human eyes. Invest Ophthalmol Vis Sci.

[24] Shepard AR, Millar JC, Pang IH, Jacobson N, Wang WH, Clark AF (2010). Adenoviral gene transfer of active human transforming growth factor-{beta}2 elevates intraocular pressure and reduces outflow facility in rodent eyes. Invest Ophthalmol Vis Sci.

[25] McDowell CM, Tebow HE, Wordinger RJ, Clark AF (2013). Smad3 is necessary for transforming growth factor-beta2 induced ocular hypertension in mice. Exp Eye Res.

[26] Swaminathan SS, Oh DJ, Kang MH, Shepard AR, Pang IH, Rhee DJ (2014). Tgf-beta2-mediated ocular hypertension is attenuated in sparc-null mice. Invest Ophthalmol Vis Sci.

[27] Logan CY, Nusse R (2004). The wnt signaling pathway in development and disease. Annu Rev Cell Dev Biol.

[28] Mao W, Millar JC, Wang WH, Silverman SM, Liu Y, Wordinger RJ, Rubin JS, Pang IH, Clark AF (2012). Existence of the canonical Wnt signaling pathway in the human trabecular meshwork. Invest Ophthalmol Vis Sci.

[29] Wang WH, McNatt LG, Pang IH, Millar JC, Hellberg PE, Hellberg MH, Steely HT, Rubin JS, Fingert JH, Sheffield VC, Stone EM, Clark AF (2008). Increased expression of the wnt antagonist sfrp-1 in glaucoma elevates intraocular pressure. J Clin Invest.

